# Subchondral fracture caused by unevenly stiffened meniscus after radiofrequency-assisted arthroscopic knee meniscectomy: A case report and review of the literature^☆^

**DOI:** 10.1016/j.ijscr.2019.10.049

**Published:** 2019-10-28

**Authors:** Kiyokazu Fukui, Akihiro Shioya, Yoshiyuki Tachi, Katsutaka Yonezawa, Hiroaki Hirata, Norio Kawahara

**Affiliations:** a Department of Orthopedic Surgery, Kanazawa Medical University, Japan; b Department of Pathology and Medical Laboratory, Kanazawa Medical University, Japan

**Keywords:** Spontaneous osteonecrosis of the knee, Postarthroscopic osteonecrosis of the knee, Subchondral fracture, Radiofrequency-assisted arthroscopic knee meniscectomy

## Abstract

**Introduction:**

Causality for postarthroscopic osteonecrosis of the knee is unknown, and related mechanisms have been poorly characterized.

**Presentation of case:**

This report describes a case of a 69-year-old man with subchondral fracture occurring after arthroscopic meniscectomy using a radiofrequency assisted shaver. The patient experienced increasingly intense knee pain 10 months after the meniscectomy. MR imaging revealed postarthroscopic osteonecrosis of the knee in the femoral medial condyle, requiring unicompartmental knee arthroplasty. A mid-coronal cut section of the resected medial femoral condyle showed a linear fracture line parallel to the subchondral bone endplate. Histopathological examination showed prominent callus formation on both sides of the fracture, comprised of reactive woven bone and granulation tissue. The middle portion of the resected medial meniscus was of uneven height, with significant stiffening of the higher side. The stiffened region of the medial meniscus corresponded to the subchondral fracture in the medial femoral condyle.

**Discussion:**

The etiology of post-arthroscopic osteonecrosis of the knee is controversial, but it seems possible that altered knee biomechanics after meniscectomy may predispose patients to osteonecrosis. The findings of the current case suggested that uneven stiffening of the meniscus caused concentration of stress that resulted in postarthroscopic subchondral fracture.

**Conclusion:**

Subchondral insufficiency fracture following arthroscopy may be underdiagnosed. Surgeons need to carefully consider the risk of subchondral fracture following uneven stiffening of the meniscus when to use radiofrequency in the debridement of a torn meniscus.

## Highlights

• Although the worsening of symptoms following knee arthroscopy in older patients is often labeled as progression of arthritic symptoms, subchondral insufficiency fracture following arthroscopy may be underdiagnosed.
• There is a possibility that uneven stiffening of the meniscus causes concentration of stress that resulted in postarthroscopic subchondral fracture.
• Surgeons should consider avoiding subsequent subchondral fracture when to use radiofrequency in the debridement of a torn meniscus.

## 1 Introduction

Spontaneous osteonecrosis of the knee (SONK) was first described in 1968 by Ahlback et al. as a “distinct clinical entity” primarily affecting older women [1]. Patients with SONK generally experience acute medial knee pain, with variable radiography showing focal osteonecrosis in the medial femoral condyle [2,3]. Many theories have been developed about the causality of spontaneous osteonecrosis of the knee [[4], [5], [6], [7], [8]]; recent research has led to recognition of subchondral fracture as a substantial contributing element in this form of osteonecrosis [[9], [10], [11], [12]]. Additionally, osteonecrosis has been documented as a rare complication of arthroscopic knee surgery, diagnosed on the basis of radiographic findings subsequent to recurrent or worsening clinical symptoms [13]. Most cases have been associated with routine meniscectomy and chondroplasty using a mechanical shaver [[14], [15], [16], [17], [18]], and cases have also been reported following laser-assisted meniscectomy [[19], [20], [21], [22]]. In this paper, we assess the characteristics of bone and meniscus that were resected from a patient during unicompartmental knee arthroplasty. The patient was previously diagnosed with subchondral fracture after arthroscopic knee meniscectomy that had been performed using a laser-assisted device. The patient provided written informed consent for publication of the case. The patient was informed that data concerning the case would be submitted for publication, and he provided consent. This work has been reported in line with the SCARE criteria [23].

## 2 Case presentation

**Fig. 1:**
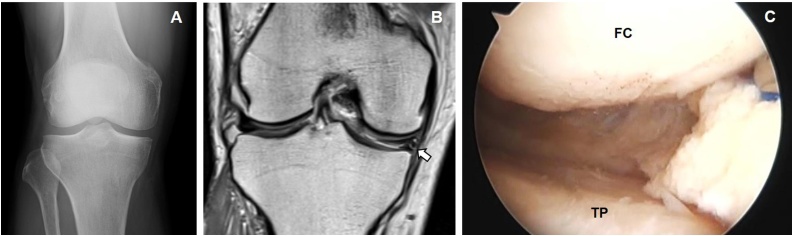
**(A)** Initial anteroposterior radiograph showed no significant findings. **(B)** T2-weighted magnetic resonance images showed a straight high intensity line indicating a horizontal tear of the medial meniscus (red arrow). **(C)** Intraoperative image of arthroscopic meniscectomy. Meniscus height is obviously uneven in between the middle portion of the meniscus and the resected posterior portion.

A 69-year-old man (height 165 cm, weight 72 kg, body mass index 26 kg/m^2^) came to our hospital with a complaint of right medial knee pain and no apparent cause. Medical history was unremarkable, and there was no history of previous trauma, alcohol use, or intra-articular steroid use. The initial physical examination showed no effusion of the right knee, full range of motion, and normal tibiofemoral alignment (femorotibial mechanical axis angle of 177°). After about 5 months of conservative treatment, including intra-articular injection (hyaluronic acid), the knee pain had worsened. We found no ligamentous instability. McMurray’s test was positive medially, with tenderness to palpation at the medial joint line. Initial radiography was normal. Magnetic resonance imaging (MRI) showed a horizontal tear in the middle and posterior segments of the medial meniscus. Articular and osseous anatomy was unremarkable (Fig. 1A,B).

**Fig. 2:**
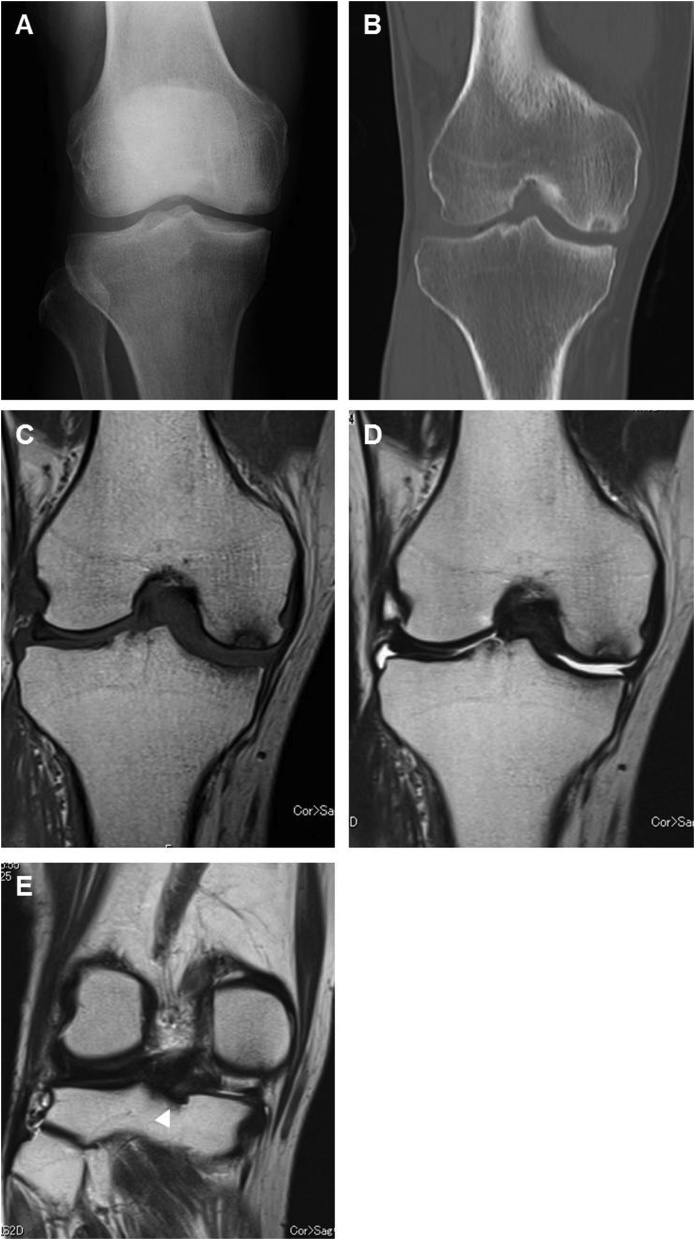
**(A)** AP radiograph 10 months after arthroscopic surgery showed focal radiolucency in the weight-bearing area of the medial femoral condyle. **(B)** Multiplanar reconstruction indicated a sclerotic band in the weight-bearing area of the medial femoral condyle. **(C)** The T1-weighted image showed diffuse low signal intensity in the medial femoral condyle, and an associated band of lower signal intensity was seen in the area of the lesion. **(D)** The T2-weighted image had an inhomogeneous area of high signal intensity in the corresponding region and showed a focal area of low signal intensity underlying the articular cartilage. **(E)** T2-weighted images generally showed that the posterior root of the medial meniscus had been preserved (arrowhead).

We conducted arthroscopic surgery of the right knee 7 months after the onset of pain. Ordinary infusion of normal saline solution was used to distend the joint. A tourniquet, inflated to 300 mmHg was used for 90 min total during the procedure. MRI findings of a horizontal tear in the middle and posterior segments of the medial meniscus were substantiated, and basket forceps and a radiofrequency assisted shaver (DYONICS ELECTROBLADE, Smith & Nephew, London, United Kingdom) were used to perform a partial medial meniscectomy (Fig. 1C). Radiographs from 10 months after the index surgery showed a small radiolucent area in the medial femoral condyle of the right knee (medial side of weight-bearing area) (Fig. 2A). Computed tomography was used to create a multiplanar reconstruction image showing a sclerotic band at the same part of the radiograph (Fig. 2B). T1-weighted MRI, performed immediately after the radiographic check-up, showed a low-intensity rounded band within the weight-bearing area of the medial femoral condyle and surrounded by a diffuse low-intensity area (Fig. 2C). T2-weighted MRI showed the same band, surrounded by an edematous lesion in the bone marrow (Fig. 2D). The weight-bearing area of the medial femoral condyle appeared to be slightly flattened. We judged that the posterior root of the medial meniscus had been preserved as described in the classification by Robertson et al. [24] (Fig. 2E), and we diagnosed stage 2 SONK based on to the classification by Koshino [25]. Thirteen months after index surgery, the patient had unicompartmental knee arthroplasty.

**Fig. 3:**
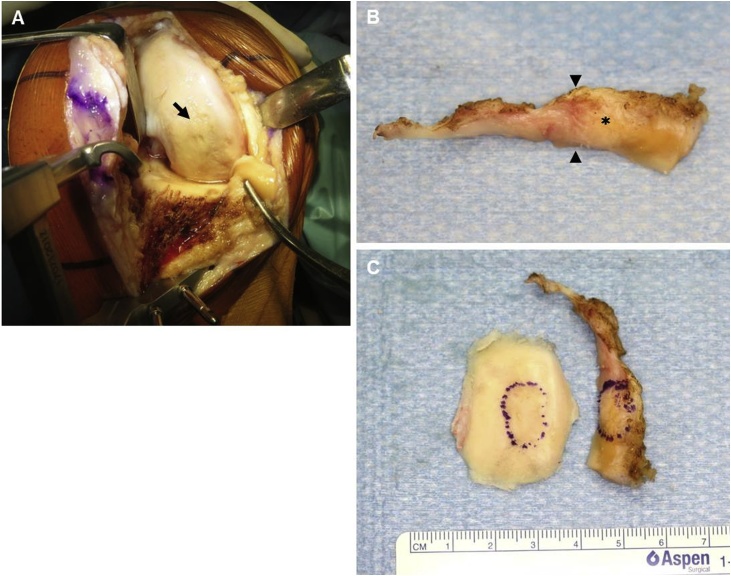
**(A)** Intraoperative findings of for the cartilage surface of the medial femoral condyle showed a mild cartilage wear but no detachment from the osteochondral lesion (arrow). **(B)** Although the posterior portion of the medial meniscus was thinned by arthroscopic meniscectomy, the posterior root was intact. The middle portion of the medial meniscus was of uneven height (black arrowheads) and the higher side (asterisk) was significant stiffer than the other portions. **(C)** The stiffened area of the medial meniscus corresponded to the subchondral fracture of the medial femoral condyle (dotted circle).

**Fig. 4:**
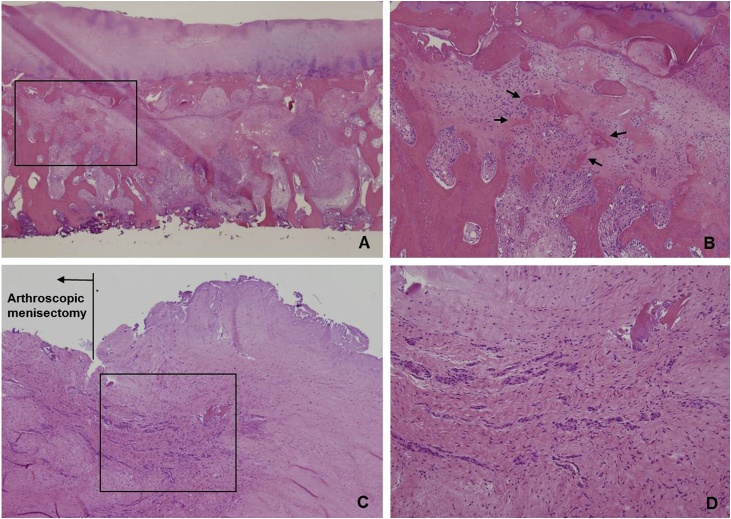
**(A)** Photomicrograph of the resected medial condyle demonstrating a compression fracture of the　medial femoral condyle with intact overlying articular cartilage. **(B)** Enlarged image of the left area (box) in Fig. 4A. Prominent callus formation (arrows) was seen, consisting of reactive woven bone and granulation tissue. We found no evidence of antecedent bone infarction such as creeping substitution or bone marrow necrosis. (Hematoxylin and eosin stain, A; ×40. B; ×100) **(C)** Photomicrograph of the resected meniscus near the area of uneven height **(D)** Enlarged image of the left area (box) in Fig. 4C; there was visible aggregation of fibroblasts and collagen fibers.

During surgery, we noted slight irregularities in the cartilage surface of the medial femoral condyle but no detachment of the osteochondral lesion (Fig. 3A). A hook test showed no obvious instability of the drawing the medial meniscus, and no posterior root tears were noted. Pinching showed the stiffened area in the middle part of the meniscus to be much harder than other parts of the resected meniscus (Fig. 3B). The subchondral fracture of the medial femoral condyle was located at just over the stiffened area of the medial meniscus (Fig. 3C). Histopathologically, prominent callus formation was seen comprising reactive woven bone and granulation tissue on both sides of the fracture. Fracture-related bone debris was focally observed on the osteochondral side of the fracture (Fig. 4A,B). There was no histopathological evidence of any antecedent bone infarction, such as creeping substitution or bone marrow necrosis. The resected meniscus showed a proliferation of fibroblasts and collagen fibers corresponding to the stiffened area (Fig. 4C,D).

## 3 Discussion

Yamamoto and Bullough reviewed histopathologic changes in 14 patients diagnosed with SONK not associated with arthroscopic surgery, and concluded that the primary event was a subchondral insufficiency fracture [10]. Robertson et al. stated that the etiology of SONK remains elusive and is probably multifactorial, but suggested the possibility that posterior medial meniscal root injury contributes to SONK development [24]. Sung et al. compared the two groups for posterior root tear and horizontal tear [26]; their findings supported Robertson’s hypothesis. In addition, Yamagami et al. demonstrated a clear association between spontaneous osteonecrosis of the knee and the presence of medial meniscus posterior root tear; SONK patients had greater medial meniscus extrusion and steeper posterior tibial slope [27]. Hussain et al. performed a comprehensive review of 26 out of 255 articles and found that 21 (80.7%) of those 26 articles implicated the role of the meniscus in the emergence of SONK, either in association with meniscal tears or with the development of SONK after meniscectomy [9]. There has been some confusion because of ambiguity associated with terms such as osteonecrosis and subchondral fracture, and this confusion may be aggravated by similarities in imaging and pathologic findings. Osteonecrosis refers to dead bone, possibly resulting from disruption of the blood supply; it is also called avascular necrosis. Recent findings suggest that SONK may misrepresent the etiology and pathogenesis of this condition, and that the term “subchondral insufficiency fractures of the knee” would be more accurate [[9], [10], [11], [12],^24^,^26^,^27^].

The condition of post-arthroscopic osteonecrosis of the knee (PAONK) was first described by Brahme et al. in 1991 [28]. Considering that arthroscopic meniscectomies are performed in large numbers worldwide, the prevalence of PAONK appears remarkably low. In patients more than 50 years of age who underwent a total of 585 arthroscopic meniscectomy, Pruès-Latour et al. found nine cases (1.5%) [29]. Santori et al. reviewed more than 2000 knee arthroscopies performed at their hospital over 10 years; they found only two documented cases of osteonecrosis (0.2%) [30]. Pape et al. reviewed 47 cases of PAONK described in the literature after arthroscopic meniscectomy [13].

The etiology of PAONK is controversial, but it seems possible that altered knee biomechanics after meniscectomy may predispose patients to osteonecrosis [31]. Increased tibiofemoral contact pressure could lead to insufficiency fracture of the cartilage and subchondral bone, potentially associated with intraosseous leakage of synovial fluid, followed by osteonecrosis [32,33]. Preexisting cartilage damage may increase permeability for the arthroscopy fluid, possibly leading to subchondral edema and consequent osteonecrosis [34]. The role of radiofrequency procedures has also been investigated in relation to the occurrence of PAONK [[34], [35], [36]]. The effect of heat on the fluid medium and the direct transfer of energy to subchondral bone were once thought to explain the mechanism of PAONK after radiofrequency treatment [[36], [37], [38], [39]]. However, other studies found that radiofrequency chondroplasty resulted in fewer instances of PAONK in patients [[40], [41], [42]]. When compared with mechanical shaving, radiofrequency debridement reduced chondrocyte death, shortened operative time, and provided a smooth joint surface that prevented irregular surface contact and resulted in fewer postoperative clinical symptoms [[43], [44], [45]]. Most of the studies concluded that there was no meaningful relationship between radiofrequency debridement and PAONK, and that postarthroscopic osteonecrosis of the knee results primarily from meniscal tears and partial meniscectomy.

We confirmed that the histopathological cause of spontaneous osteonecrosis of the knee lesion was subchondral fracture, not primary osteonecrosis. Some reports have suggested an association between posterior root tear of the medial meniscus and spontaneous osteonecrosis of the knee. However, in this case we found no posterior root tear. Instead, we noted uneven height and radiofrequency-induced stiffening in the resected margin at the middle segments of the medial meniscus. The subchondral fracture in our patient was in the medial center portion of the medial condyle; immediately below was the stiffened swollen meniscus in an upright position. In this case, we speculate that the unevenly stiffened meniscus may have concentrated stress on the fractured lesion, resulting in subchondral fracture. This phenomenon may be similar to that reported by Fukui et al., who noted inversion of the acetabular labrum in the initial stage of rapidly destructive hip osteoarthritis and proposed that this inversion may relate to subchondral fracture of the femoral head due to the concentration of stress on the subchondral bone [[46], [47], [48], [49]].

To the best of our knowledge, this is the first paper to discuss the relationship between subchondral fracture after arthroscopic meniscectomy and uneven stiffened meniscus due to radiofrequency debridement of the torn meniscus. In our experience, the worsening of symptoms following knee arthroscopy in older patients is very often labeled as progression or aggravation of arthritic symptoms. As a result, subchondral insufficiency fracture following arthroscopy may be underdiagnosed. To avoid subsequent subchondral fracture, surgeons need to carefully consider when to use radiofrequency in the debridement of a torn meniscus.

## Sources of funding

None.

## Ethical approval

Case reports are exempt from the need of IRB approval in our institute.

## Consent

Written informed consent was obtained from the patient for publication of this case report and accompanying images. A copy of the written consent is available for review by the Editor-in-Chief of this journal on request.

## Author contribution

**Conceptualization, Writing of manuscript, Literature review:** Kiyokazu Fukui.

**Data collections:** Kiyokazu Fukui, Katsutaka Yonezawa, Hiroaki Hirata, Yoshiyuki Tachi, Akihiro Shioya.

**Data analysis:** Kiyokazu Fukui, Akihiro Shioya.

**Reviewing of the final version of the manuscript:** Kiyokazu Fukui, Norio Kawahara.

## Registration of research studies

Not applicable.

## Guarantor

Kiyokazu Fukui.

## Provenance and peer review

Not commissioned, externally peer-reviewed.

## Declaration of Competing Interest

None.
